# Association between ABO blood groups and susceptibility to COVID-19: profile of age and gender in Iraqi patients

**DOI:** 10.1186/s43042-020-00115-y

**Published:** 2020-12-17

**Authors:** Ali H. Ad’hiah, Maha H. Abdullah, Mustafa Y. Alsudani, Rasool M. S. Shnawa, Ali J. R. Al-Sa’ady, Risala H. Allami, Khawla I. Misha’al, Iftikhar A. Jassim, Estabraq A. Taqi

**Affiliations:** 1 Tropical-Biological Research Unit, College of Science, University of Baghdad, Al-Jadriya, Baghdad, Iraq; 2 College of Biotechnology, Al-Nahrain University, Baghdad, Iraq; 3 Basrah Health Office, Basrah, Ministry of Health and Environment, Baghdad, Iraq; 4 Alforat Hospital, Baghdad, Ministry of Health and Environment, Baghdad, Iraq; 5 Biotechnology Department, College of Science, University of Baghdad, Baghdad, Iraq

**Keywords:** COVID-19, ABO blood groups, Age, Gender, Logistic regression analysis

## Abstract

**Background:**

A case-control study was performed to examine age, gender, and ABO blood groups in 1014 Iraqi hospitalized cases with Coronavirus disease 2019 (COVID-19) and 901 blood donors (control group). The infection was molecularly diagnosed by detecting coronavirus RNA in nasal swabs of patients.

**Results:**

Mean age was significantly elevated in cases compared to controls (48.2 ± 13.8 *vs*. 29.9 ± 9.0 year; probability [*p*] < 0.001). Receiver operating characteristic analysis demonstrated the predictive significance of age in COVID-19 evolution (Area under curve = 0.858; 95% CI: 0.841 – 0.875; *p* < 0.001). Males outnumbered females in cases (60.4 *vs*. 39.6%) and controls (56 *vs*. 44%). Stratification by age group (< 30, 30 – 39, 40 – 49 and ≥ 50 years) revealed that 48.3% of cases clustered in the age group ≥ 50 years. ABO blood group analysis showed that group A was the most common among cases, while group O was the most common among controls (35.5 and 36.7%, respectively). Blood groups A (35.5 *vs*. 32.7; corrected *p* [*pc*] = 0.021), A+AB (46.3 *vs*. 41.7%; *pc* = 0.021) and A+B+AB (68.0 *vs*. 63.3%; *pc* = 0.007) showed significantly elevated frequencies in cases compared to controls. Logistic regression analysis estimated odds ratios (ORs) of 1.53 (95% confidence interval [CI]: 1.16 - 2.02), 1.48 (95% CI: 1.14 - 1.93) and 1.50 (95% CI: 1.17 - 1.82) for blood groups A, A+AB and A+B+AB, respectively. Blood group frequencies showed no significant differences between age groups of cases or controls. Regarding gender, male cases were marked with increased frequency of group A (39.9 *vs*. 28.9%) and decreased frequency of group O (25.9 *vs*. 41.0%) compared to female cases. Independent re-analysis of ABO blood groups in male and female cases demonstrated that group A was increased in male cases compared to male controls (39.9 *vs*. 33.1%; OR = 1.65; 95% CI: 1.24 - 2.21; *pc* = 0.006). On the contrary, no significant differences were found between females of cases and controls.

**Conclusions:**

The study results indicated that blood group A may be associated with an increased risk of developing COVID-19, particularly in males.

## Background

Coronavirus disease 2019 (COVID-19) is a pandemic respiratory infection caused by a novel coronavirus termed severe acute respiratory syndrome coronavirus 2 (SARS-CoV-2). It has been originated in the city of Wuhan (Central China) and showed a global spread with accelerating rates associated with increasing morbidities and mortalities that seriously impacted public health worldwide [1]. The disease has spread across 219 countries and up to the 15^th^ of November 2020, a total of 54,589,646 confirmed COVID-19 cases have been reported with 2.4% death rate (1,322,072 cases). The corresponding figures for Iraq are 519,152 cases with 2.2% death rate (11,670 cases). These figures ranked Iraq as the 20^th^ among other world countries [2].

Up-to-date, there has been no effective therapeutic medicine or vaccine that aid in controlling this communicable disease. Therefore, it is essential to understand risk factors that may contribute to evolution of COVID-19. Epidemiological reviews have depicted that all populations are generally at risk to develop the infection. However, some susceptibility factors have been suggested to increase the risk of COVID-19, and age may be among these factors. Although all age groups are at risk of developing the disease, elderly people are more likely to be severely affected by COVID-19. Children have been observed to have less severe clinical symptoms, but critical illness has been found in those younger than 1 year old. It has also been suggested that infants may be at high risk of severe respiratory failure due to COVID-19 [3–5]. Gender has been further described as a risk factor for COVID-19, and males tended to have a more severe disease than females. Mortality rates are also higher in males than in females [5, 6]. Some biological determinants of the host have also been considered as prominent markers associated with susceptibility to COVID-19. Among these determinants, ABO blood groups have recently been introduced as important predisposing factors in the development of infection [7–15].

ABO blood groups and since their first discovery by Karl Landsteiner in the beginning of the last century have potentiated clinical, immunological and anthropological investigations. Beyond their role in transfusion medicine, alleles and phenotypes of ABO system show racial- and population-based variations [16, 17]. Further, the risk of developing some diseases is influenced by alleles or phenotypes of ABO blood groups; for instance, blood group O in peptic ulcer, I*A allele in chronic myeloid leukemia, blood groups A, B and AB in SARS, and blood groups B and AB in pathogenic enteric infections. In infectious diseases, host susceptibility may be related to differences in antigen expression of blood groups, which can serve as receptors and/or co-receptors for the infectious agent [18, 19].

In line with these presentations, this study aimed to investigate the genetic association of ABO blood groups (A, B, AB and O) with risk of COVID-19 in Iraqi patients. In a previous issue of this journal, we suggested that group A was associated with an increased risk of death in COVID-19 cases, while AB may be a susceptibility biomarker [20]. However, the study was limited by low sample size of patients (300 cases). In order to gain a better understanding of these biomarkers in risk of disease, the sample size was increased to 1014 cases. This sample size allowed for ABO blood groups to be re-analyzed in COVID-19 cases with emphasis on age and gender. Thus, the confounding effect of age and gender on the association between ABO blood groups and evolution of COVID-19 was evaluated.

## Methods

### Patients and controls

During the period from May 31 to July 31, 2020, a case-control study was conducted to examine the genetic association of ABO blood groups (A, B, AB and O) with COVID-19 in Iraqi patients. A total of 1014 patients were enrolled in the study. They were admitted to hospitals in two major Iraqi cities (Baghdad and Basrah). The infection was molecularly diagnosed by detecting the coronavirus RNA in nasal swabs of patients using a detection kit for 2019 novel coronavirus (2019-nCoV) RNA (PCR-Fluorescence Probing; Da An Gene Co., Ltd. of Sun Yat-sen University; China). Included patients are those with sign and symptoms of respiratory disease and tested positive for COVID-19 nucleic acid. Patients with respiratory complications and tested negative for the virus RNA were excluded from the study. Data on age, gender, and blood group for each patient were obtained from hospital records. A control sample of 901 individuals was also included in the study. They were blood donors and their anti-viral tests at the Central Blood Banks (Baghdad and Basrah) were negative. The protocol of study was approved by the Ethics Committees at the Iraqi Ministry of Health and Environment, and the guidelines issued by this committee were followed.

### Statistical analysis

The phenotypes of ABO blood groups (A, B, AB and O) were given as numbers and percentage frequencies. Allele frequencies and Hardy-Weinberg equilibrium (HWE) were estimated using the software S2 ABOestimator (http://webpages.fc.ul.pt/~pjns/Soft/ABOestimator). Genetic association of ABO blood group with COVID-19 was assessed using logistic regression analysis (adjusted for age and gender) and group O was the reference category. The association was expressed as odds ratio (OR) and 95% confidence interval (CI). Ages were given as mean ± standard deviation (SD), and significant differences between means were assessed by one-way analysis of variance (ANOVA) followed by either least significant difference (LSD) or Duncan's multiple range post-hoc test. To assess the role of age in predicting disease, receiver operating characteristic (ROC) analysis was performed to estimate area under curve (AUC) occupied by age. In this analysis, age was the test variable, while COVID-19 cases were the state variable. A probability (*p*)-value ≤ 0.05 was considered statistically significant after applying Bonferroni correction (*pc*). These analyses were carried out using the statistical package IBM SPSS Statistics 25.0 (Armonk, NY: IBM Corp.). G*Power software was used to determine power of sample size.

## Results

### Power of sample size

At alpha probability of 0.05 and effect size of 0.1, the power of sample size was 0.94. Accordingly, the representation of sample size was statistically validated.

### Age and gender distributions

**Fig. 1:**
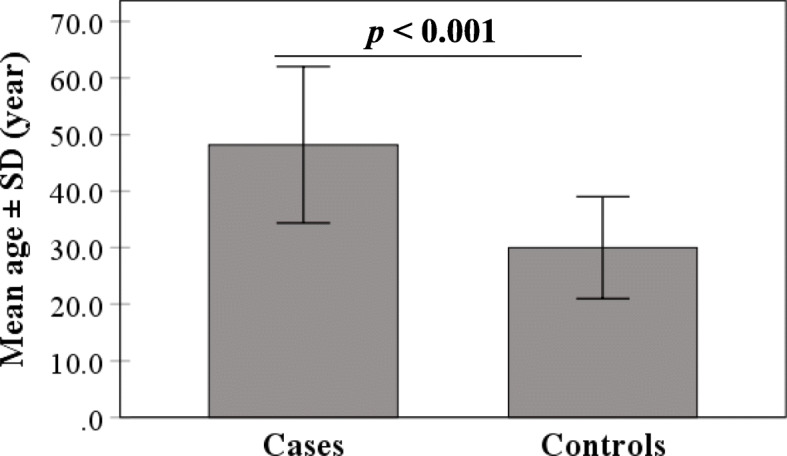
Mean age of COVID-19 cases and controls

**Fig. 2:**
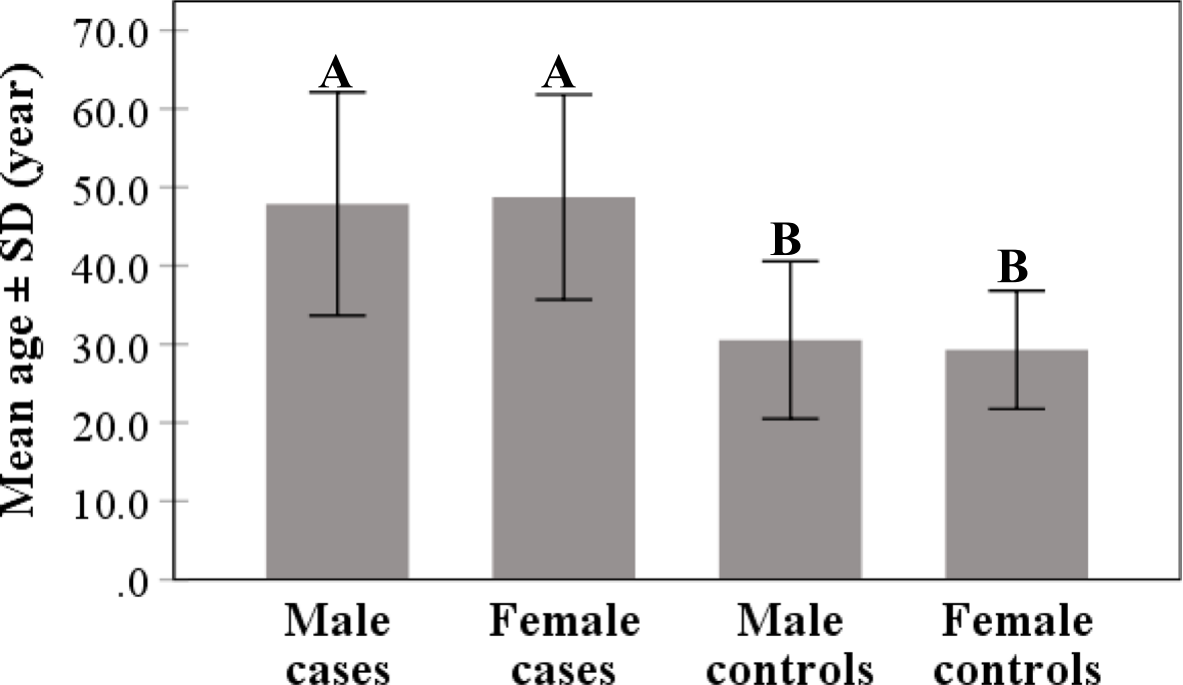
Mean age of COVID-19 cases and control distributed according to gender. Different letters denote significant difference between means of bars (*p* < 0.001), while similar letters denote no significant difference (*p* > 0.05) (Duncan’s multiple range test)

**Fig. 3:**
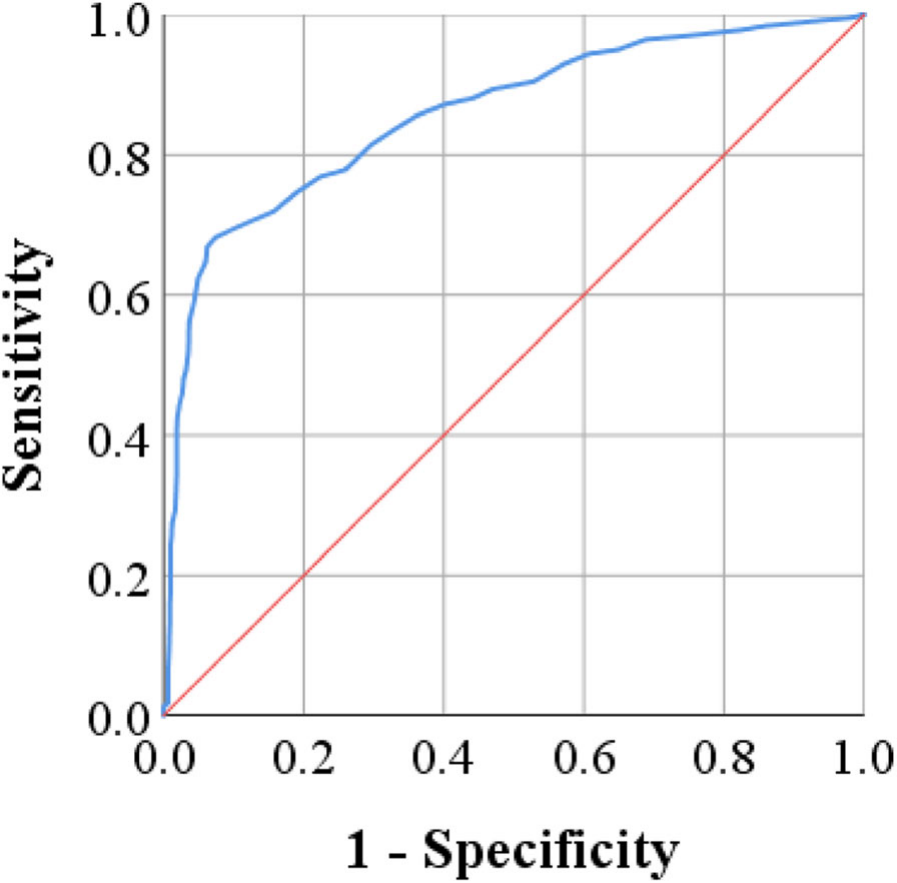
Receiver operating characteristic (ROC) analysis of age in COVID-19 cases (area under curve = 0.858; 95% confidence interval 0.841–0.875; *p* < 0.001)

Mean age was significantly elevated in COVID-19 cases compared to controls (48.2 ± 13.8 *vs*. 29.9 ± 9.0 year; *p* < 0.001) (Fig. 1). A similar observation was made in male (47.8 ± 14.3 *vs*. 30.5 ± 10.0 year; *p* < 0.001) and female (48.7 ± 13.1 *vs*. 29.3 ± 7.5; year; *p* < 0.001) cases, while there was no significant difference between males and females regarding mean age in patients or controls (Fig. 2). ROC analysis demonstrated the predicting significance of age in evolution of COVID-19. The estimated AUC was 0.858 (95% CI: 0.841 – 0.875; *p* < 0.001) (Fig. 3).

**Fig. 4:**
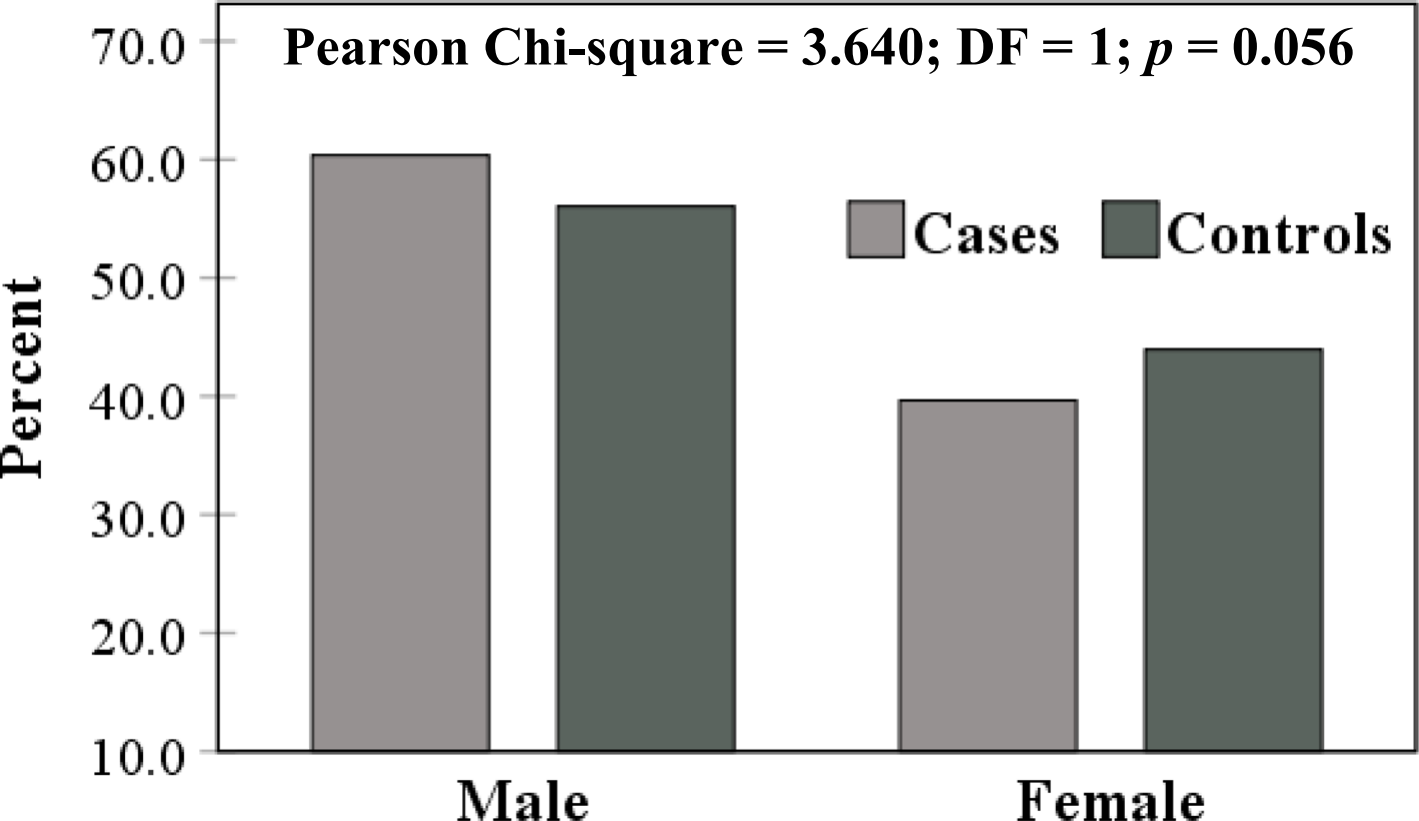
Gender distribution of COVID-19 cases and controls

**Fig. 5:**
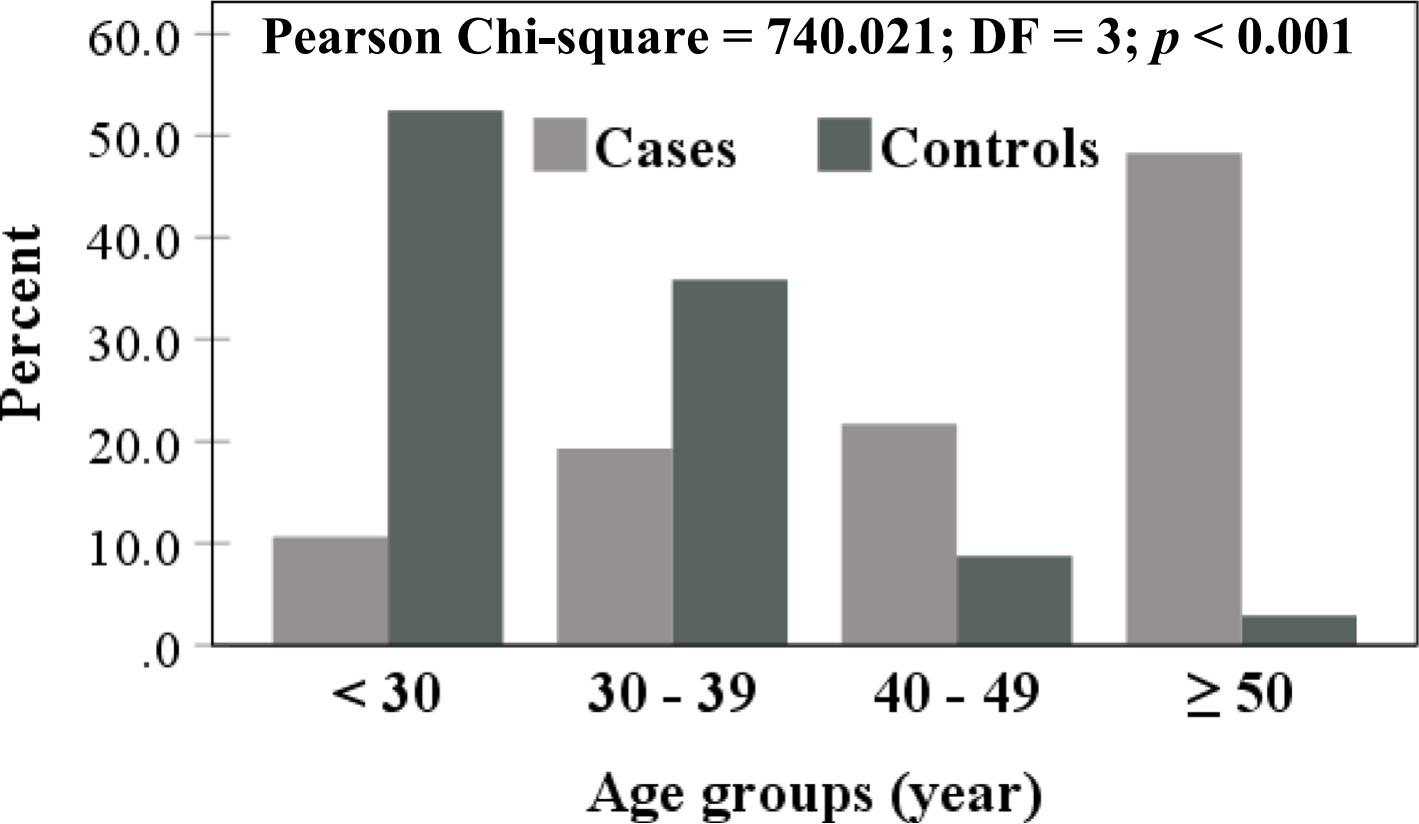
COVID-19 cases and control distributed according to age groups

**Fig. 6:**
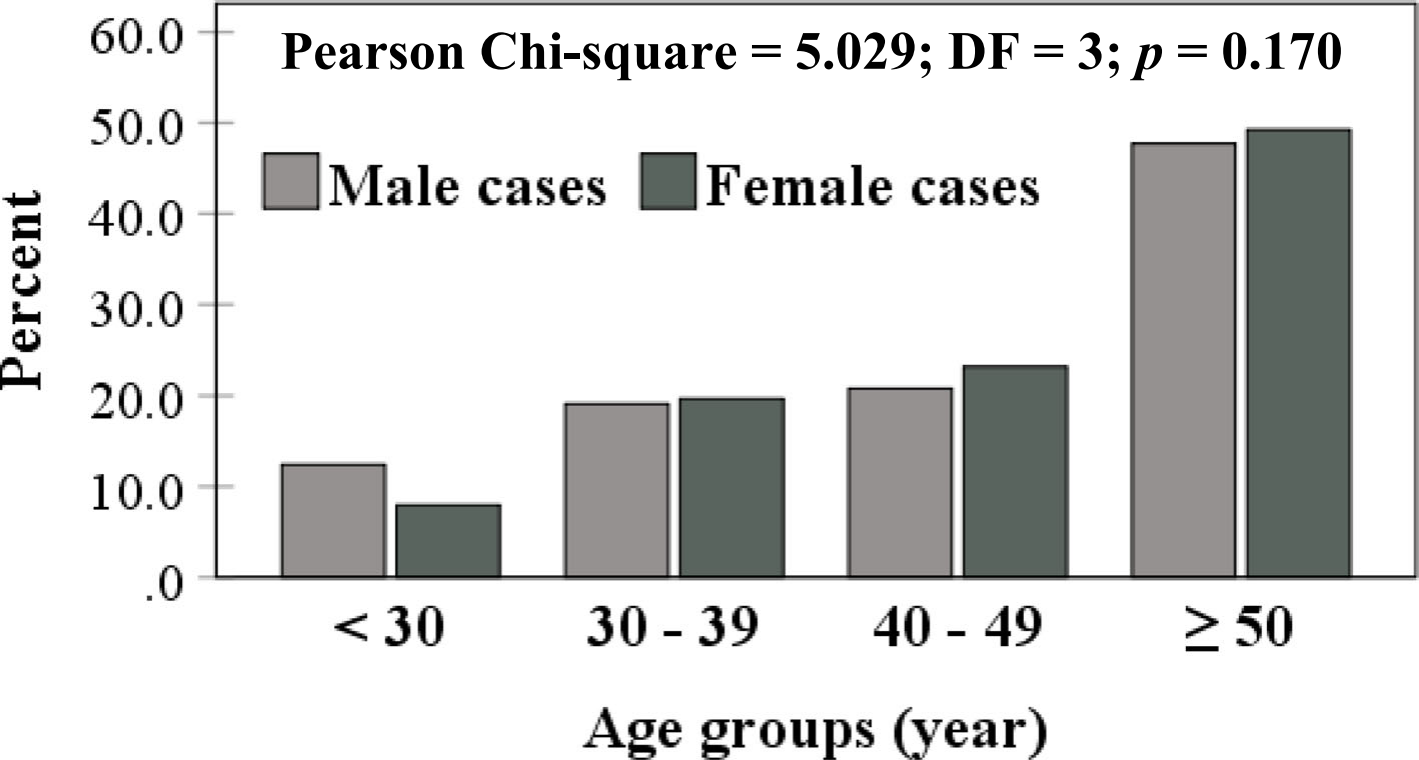
COVID-19 cases distributed according to age groups and gender

Males outnumbered females in cases (60.4 *vs*. 39.6%) and controls (56 *vs*. 44%), but no significant difference was recorded (*p* = 0.056) (Fig. 4). However, age grouping (< 30, 30 – 39, 40 – 49 and ≥ 50 years) revealed significant variation between cases and controls (*p* < 0.001). Approximately, 50% of COVID-19 cases clustered in the age group ≥ 50 years (48.3%). On the contrary, more than 50% of controls were below the age of 30 years (Fig. 5). Distribution of age groups in male and female cases showed no significant differences (Fig. 6).

### ABO blood group distributions in patients and controls

ABO blood group analysis showed that group A was the most common among cases, while blood group O was the most common among controls (35.5 and 36.7%, respectively). Blood groups A (35.5 *vs*. 32.7; *pc* = 0.021), A+AB (46.3 *vs*. 41.7%; *pc* = 0.021) and A+B+AB (68.0 *vs*. 63.3%; *pc* = 0.007) showed significantly increased frequencies in COVID-19 cases compared to controls. The estimated ORs of these variations (logistic regression analysis: groups A, A+AB or A+B+AB *vs*. O) were 1.53 (95% CI: 1.16 - 2.02), 1.48 (95% CI: 1.14 - 1.93) and 1.50 (95% CI: 1.17 - 1.82), respectively (Table 1).

**Table 1:** ABO blood group distributions in COVID-19 cases and controls

Phenotype	*N* (%)		OR	95% CI	*p*	pc
	Controls (*N* = 901)	Cases (*N* = 1014)				
O	331 (36.7)	324 (32.0)	Reference			
A	295 (32.7)	360 (35.5)	1.53	1.16–2.02	**0.003**	**0.021**
B	194 (21.5)	221(21.8)	1.16	0.91–1.49	0.234	1.000
AB	81 (9.0)	109 (10.7)	1.33	0.88–2.02	0.180	1.000
A + B	489 (54.3)	581 (57.3)	1.21	1.00–1.47	0.053	0.371
A + AB	376 (41.7)	469 (46.3)	1.48	1.14–1.93	**0.003**	**0.021**
B + AB	275 (30.5)	330 (32.5)	1.46	1.10–1.94	0.080	0.480
A + B + AB	570 (63.3)	690 (68.0)	1.50	1.17–1.82	**0.001**	**0.007**

*OR* odds ratio, *CI* confidence interval, *p* logistic regression probability adjusted for age and gender, *pc* Bonferroni corrected *p*. Significant *p* value is bold-marked

### Allele frequencies of ABO blood groups

Allele frequencies of ABO blood groups showed no significant deviation from HWE in COVID-19 cases or controls. However, the cases were characterized by increased frequencies of *p*[A] and *q*[B] alleles (0.265 and 0.178, respectively) compared to controls (0.235 and 0.166, respectively). Conversely, the frequency of *r*[O] allele decreased in cases (0.557 *vs*. 0.599) (Table 2).

**Table 2:** Estimated frequencies of ABO blood group alleles in COVID-19 cases and controls

Group	Allele frequency (standard deviation)			Hardy-Weinberg equilibrium		
	*p*[A]	*q*[B]	*r*[O]	Log likelihood	Chi-square	*p*
Cases	0.265 (0.010)	0.178 (0.009)	0.557 (0.012)	− 1323.8	3.078	0.075
Controls	0.235 (0.011)	0.166 (0.009)	0.599 (0.013)	− 1155.1	2.535	0.111

*p* probability

### Age and gender distributions of ABO blood groups

Frequencies of blood groups A, B, AB and O showed no significant differences between age groups (< 30, 30 – 39, 40 – 49 and ≥ 50 years) in COVID cases or controls (Table 3). With respect to gender, these frequencies showed significant differences between male and female cases (*p* < 0.001). Male cases were characterized by increased frequency of group A (39.9 *vs*. 28.9%) and decreased frequency of group O (25.9 *vs*. 41.0%) compared to female cases. These differences between males and females were not observed in controls (Table 4). Therefore, ABO blood groups were re-analyzed independently in males and females. The analysis revealed that group A frequency elevated in male cases compared to male controls (39.9 *vs*. 33.1%), while group O frequency decreased (25.9 *vs*. 35.6%). Logistic regression analysis (group A *vs*. group O) estimated an OR of 1.65 (95% CI: 1.24 - 2.21), and the difference was significant (*pc* = 0.006). On the contrary, there were no significant differences between females of cases and controls (Table 5).

**Table 3:** ABO blood groups in COVID-19 cases and controls distributed according to age groups

Blood group	*N* (%)							
	Cases (*N* = 1014)				Controls (*N* = 901)			
	< 30 (*N* = 108)	30–39 (*N* = 196)	40–49 (*N* = 220)	≥ 50 (*N* = 490)	< 30 (*N* = 473)	30–39 (*N* = 323)	40–49 (*N* = 79)	≥ 50 (*N* = 26)
A	43 (39.8)	68 (34.7)	79 (35.9)	170 (34.7)	162 (34.2)	103 (31.9)	26 (32.9)	4 (15.4)
B	22 (20.4)	53 (27.0)	47 (21.4)	99 (20.2)	105 (22.2)	72 (22.3)	14 (17.7)	3 (11.5)
AB	8 (7.4)	19 (9.7)	21 (9.5)	61 (12.4)	38 (8.0)	35 (10.8)	4 (5.1)	4 (15.4)
O	35 (32.4)	56 (28.6)	73 (33.2)	160 (32.7)	168 (35.5)	113 (35.0)	35 (44.3)	15 (57.7)
*p*	0.566				0.131			

*p* Pearson’s chi-squared probability

**Table 4:** ABO blood groups in COVID-19 cases and controls distributed according to gender

Blood group	*N* (%)			
	Cases (*N* = 1014)		Controls (*N* = 901)	
	Male (*N* = 612)	Female (*N* = 402)	Male (*N* = 505)	Female (*N* = 396)
A	244 (39.9)	116 (28.9)	167 (33.1)	128 (32.3)
B	140 (22.9)	81 (20.1)	111 (22.0)	83 (21.0)
AB	69 (11.3)	40 (10.0)	47 (9.3)	34 (8.6)
O	159 (25.9)	165 (41.0)	180 (35.6)	151 (38.1)
*p*	**< 0.001**		0.886	

*p* Pearson’s chi-squared probability. Significant *p* value is bold-marked

**Table 5:** Regression analysis of ABO blood groups in male and female COVID-19 cases

Comparison	Blood group	OR	95% CI	*p*	pc
Male cases vs. male controls	O	Reference			
	A	1.65	1.24–2.21	**0.001**	**0.006**
	B	1.43	1.03–1.98	**0.037**	1.000
	AB	1.66	1.08–2.55	**0.024**	0.144
Female cases vs. female controls	O	Reference			
	A	0.83	0.59–1.16	0.306	1.000
	B	0.89	0.61–1.30	0.565	1.000
	AB	1.08	0.65–1.79	0.797	1.000

*OR* odds ratio, *CI* confidence interval, *p* logistic regression probability adjusted for age and gender, *pc* Bonferroni corrected *p*. Significant *p* value is bold-marked

## Discussion

This study demonstrated that age, gender and ABO blood groups might be important risk factors for COVID-19 in Iraqi patients. The mean age of patients approached the fifth decade (48.2 ± 13.8 year), and 48.3% of patients were classified in the age group ≥ 50 years. These findings strongly suggest that individuals aged 50 years and older are at greater risk to develop COVID-19. Previous studies have likewise reported that elderly are more susceptible to COVID-19 than younger adults. Further, severity and outcome of disease largely depend on age of patient. Most of hospitalized COVID-19 cases (80%) were aged 65 years and older and tended to have a higher risk of death (23-fold) compared to younger patients [4, 21–23]. Although the elderly are at a greater risk of some comorbidities (cardiovascular diseases, diabetes, obesity and respiratory system diseases), immunological dysfunctional abnormalities have been introduced as risk factors for the evolution of COVID-19 in the elderly population [23]. Two immunological outcomes have been correlated with aging; inflamm-aging and immunosenescence. In inflamm-aging, elevated levels of peripheral pro-inflammatory mediators; for instance, interleukin (IL)-1β, IL-6 and tumor necrosis factor alpha (TNF-α), have been reported. Their elevations may drive the development and maintenance of immunosenescence, which refers to aging-related immunological changes that may have detrimental effects [24, 25]. Probably, most diseases affecting the elderly (including COVID-19) are due to inflamm-aging and immunosenescence, as they contribute to what has been termed a cytokine storm. The cytokine storm is described as life-threatening organ-dysfunction syndrome due to abnormal host immune response against infectious pathogens. Dyspnea, hypoxemia and inflammation in major organs (the lungs, kidneys, heart, liver and brain) are prominent outcomes of the cytokine storm [23, 26]. In severe COVID-19 cases, it has been reported that vascular inflammation is a substantial cause of microvascular injury and thrombosis due to complement-associated activation [27]. Further immunological changes have been associated with aging. They included declined generation of CD3+ T cells, increased CD4/CD8 T cells ratio, increased regulatory T cells (Treg), decreased B lymphocytes and upregulated expression of toll-like receptors (TLRs). These consequences may contribute to the poor outcomes in elderly COVID-19 patients [28–30].

Gender can also be considered a predisposing factor for COVID-19, and males may be more susceptible to the disease than women. Among the 1014 confirmed cases of COVID-19, the proportion of males was higher than that of females (60 *vs*. 40%) and the male:female ratio was 1.5:1.0. Consistent with these findings, a Brazilian study reported that 57.5% of 67,180 cases were males [31]. On the contrary, most of epidemiological reviews and meta-analysis studies reported comparable rates of COVID-19 between males and females, but males tended to have higher fatality and mortality rates than females [5, 6, 32]. Vulnerability of men for worse outcomes of COVID-19 is probably due to gender-based immunological differences between men and women, and this may impact the woman ability to resist infections including COVID-19 [33]. Sex hormones may mediate these variations between men and women in the susceptibility to COVID-19. Experimental data demonstrated that female mice treated with an estrogen receptor antagonist showed increased mortality rates due to SARS-CoV infection. Thus, estrogen receptor signaling has been considered a critical factor in protecting female mice from the infection [34]. The gender disparity in morbidity and mortality rates among COVID-19 patients may also be related to sex-biased differences in the lung expression of angiotensin-converting enzyme 2 (ACE2), which serves as a receptor for COVID-19 entry into cells [35]. The gene encoding ACE2 is mapped to chromosome X, and its expression is also influenced by sex hormones [36]. Collectively, these findings may explain gender drive modification in the COVID-19 outcomes.

Besides age and gender, this study indicates that ABO blood group determinants can be considered biomarkers of susceptibility to COVID-19 infection, and groups A, A + B, and A + B + AB have been associated with a significantly increased risk. In line with these results, Chinese and American studies reported that group A frequency significantly elevated in COVID-19 patients compared to controls while group O frequency significantly decreased [9, 10, 12, 14, 15].. Further, a genome-wide association study was conducted at seven hospitals in the Italian and Spanish centers of the SARS-CoV-2 epidemic in Europe, and the analysis confirmed that group A was associated with a high risk of developing COVID-19, while group O had a protective effect compared to other blood groups [37].. In a study from China (Wuhan city), the frequency of group A was significantly increased in COVID-19 patients compared to controls, while groups B, AB, and O frequencies showed no significant differences [9].. A Spanish study suggested a lower susceptibility to COVID-19 for group O, while a higher risk of complications was found in group B patients [13]. Canadian data indicated that group A or AB was associated with critical illness of COVID-19 compared to group O or B [38]. In a previous Iraqi study conducted by our group, susceptibility to COVID-19 was associated with group AB in patients from Baghdad, while group A was associated with an increased risk of death [20]. In an Iranian study, no association was found with group A, but a significant decrease in group O frequency was recorded [7]. On the contrary, no association between ABO blood groups and COVID-19 was found in French and Spanish patients [8, 13]. However, in a meta-analysis study, it was emphasized that individuals of group A are at greater risk of developing COVID-19 while those of group O were at lower risk [11]. Further meta-analysis suggested that individuals of group A may be more susceptible to COVID-19, while individuals of group O may have a lower risk of developing the disease. However, the analysis also showed no relationship between ABO blood groups and severity of COVID-19 [39].

Although some inconsistent results have been reported, most studies agree that ABO blood groups are of particular significance with regard to their association with susceptibility to COVID-19, but the molecular mechanism underlying this association has not been well described. It has been hypothesized that the blood group impact on susceptibility to COVID-19 may depend on a differential clustering of the virus glycoprotein receptors on host cell surface, induced by ABO(H) determinants through interactions (carbohydrate-carbohydrate) with the glycan motif of these receptors, and this may interfere with the binding of virus and its entrance to target cells [40]. The carbohydrate structures of ABO(H) blood groups are not restricted to the surface of red blood cells, and other cells and tissues express these structures; for instance, lymphocytes, endothelial cells, platelets, gastric mucosa and bone marrow. Further, blood group antigens are present in secretions (i.e. saliva) of about 80% of individuals (ABO secretors) [41]. Therefore, their involvement in physiological and pathological processes may be expected during viral, bacterial and parasitic infections. Further evidence depicts that covalently-linked ABO(H) structures are found in some plasma glycoproteins; for instance, von Willebrand factor (VWF), and factor VIII (FVIII). It has been demonstrated that non-O individuals have significantly higher expression of endothelial cell-associated VWF protein compared to individuals of group O. The VWF expression was associated with pulmonary vascular endothelial cells and this expression was influenced by the ABO determinants [42]. In this context, elevated circulating levels of VWF and FVIII have been demonstrated in patients with severe COVID-19 pneumonia, and this may indirectly link ABO blood groups with susceptibility to COVID-19 [43]. Similar to COVID-19, group A has been associated with severe malaria, while individuals with group O are less susceptible to the infection. Further, group A individuals are more likely to have debilitated aging than those of group O [40].

To gain further understanding of the ABO-COVID-19 association, ABO blood groups were analyzed in COVID-19 patients and controls after stratification according to age group (< 30, 30-39, 40-49 and 49 50 years) and gender. In the analysis of age groups, frequencies of groups A, B, AB, and O showed no significant differences between age groups of patients or controls. Therefore, the study suggests that the ABO-COVID-19 association may not be influenced by age. A similar conclusion was reached through a Chinese study, in which the COVID-19 cases were divided into two age groups (< 40 and ≥ 40 years), and ABO blood group frequencies showed no significant differences between the two groups [12]. However, a further Chinese study reported a significantly increased frequency of group A and a significantly decreased frequency of group O in patients aged ≥ 60 years compared to patients in the age groups < 40 and 40-59 year [10].

With respect to gender, ABO blood group frequencies showed significant differences between male and female COVID-19 cases, while no significant differences were recorded between male and female controls. A re-analysis of gender-based association revealed that males of group A were more susceptible to COVID-19 than females of group A. In fact, female patients showed no significant association with ABO blood groups. These findings propose a predisposing role for group A to develop COVID-19 in males. In line with these results, a Chinese study reported that group A was encountered more frequently in male patients than in female patients [10]. On the contrary, another Chinese study reported that group A is a risk factor for COVID-19 in females but not in males. However, the study was based on low sample size of patients and controls (105 and 103, respectively) [9]. Also, no gender-related differences were seen in ABO blood groups among COVID-19 patients in two studies from China and Spain [12, 13].

## Conclusions

The study results indicated that blood group A may be associated with an increased risk of developing COVID-19, particularly in males.

## Data Availability

The datasets used and/or analyzed during the current study are available from the corresponding author on reasonable request.

## Abbreviations

ACE2: Angiotensin-converting enzyme 2
ANOVA: Analysis of variance
AUC: Area under curve
CI: Confidence interval
COVID-19: Coronavirus disease 2019
HWE: Hardy-Weinberg equilibrium
LSD: Least significant difference
OR: Odds ratio
*p*: Probability
*pc*: Bonferroni corrected *p*
ROC: Receiver operating characteristic
SARS-CoV-2: Severe acute respiratory syndrome coronavirus
SD: Standard deviation
VWF: von Willebrand factor
